# Enteric Fever in Campaigning

**Published:** 1901-08-10

**Authors:** 


### Editor's Letter-Box.

[Our Correspondents are reminded that prolixity is a great bar to publication, and that brevity of style and conciseness of statement greatly facilitate early insertion.]

### ENTERIC FEVER IN CAMPAIGNING.

Dr. Leigh Canney, referring to an article which appeared in "The Hospital" under the above heading on July 27th, has written a letter for the whole of which we regret we are unable to find room. He maintains again with much force the thesis of his pamphlet on "Typhoid in Armies," to the effect that the essential cause of typhoid epidemics in armies is usually contaminated water, and says that in soils (of fever areas) in which it might be expected to be present the bacillus typosus is never found; that it is extremely difficult for the typhoid bacillus to live in soil, and that if much moisture exists and a temperature above 66° Fahr. it will only live some hours; and that the action of light and total drying on the superficial layers of infected dust are so specially deadly to this bacillus as to account for the fact that, though it may be partly dried in the laboratory, it has not been driven through the air and recovered. He then continues:—

"The fact that there is no accepted record of an air-borne epidemic of tyyhoid is in conformity with the scientific work done and with all our knowledge of the life history of the bacillus.

"Before concluding I will give an example of what I mean by stating that 'both these agencies (flies and dust) can only act where no proper sanitary precautions are taken.' At Assouan in 1884, 1885, and 1886 the British Army had several hundreds of men encamped at selected sites at least 200 miles from the enemy. They knew the water should be protected and they made occasional attempts to do so and also to boil it. They had big epidemics each year, culminating, in 1886, with 276 cases of typhoid and 97 deaths. The medical officer at Assouan Hospital reported 'its prevalence must, in my opinion, be chiefly owing to climatic causes, and not to the insanitary condition of the camps or of the water supply'; and the P.M.O. of the Frontier Field Force further reported: 'The water supply, too, was often taken for convenience from the river's bank, and the recommendations as to boiling and filtering were not effectually carried out; but I do not think that defects of drainage, latrines, or water supply were the most important factors in the spread of the disease.' That is, these officers believed in air-borne typhoid spread by the large number of flies and much dust.

"If we take the same camp sites twelve years later we find the Civil camp of Sir John Aird's great Barrage works is situated on one of these sites, and large health resort hotels on the others. These works are exposed to clouds of dust and flies, they average some 7,000 natives and 1,000 or more Europeans, working in the bed of the river by day, and crowded in the camps by night. During three years not a single case of typhoid has originated locally, though fresh workmen from Alexandria have on six occasions brought typhoid to the camp. The trenches, at suitable places, are exposed to flies and wind. There is, however, one great difference between this camp and the military camps; in the former the prevention of typhoid is based on the water-borne theory, in the latter it was not thought that the water supply was 'the most important' cause.

"In the first year of these works I was consulted by Sir John Aird to draw up a report on the means of protecting his camps of workmen in Egypt. Amongst other questions, I attached special importance to the water supply. Of two methods suggested Sir John Aird adopted that I considered best, and in so doing, he has achieved sanitary results in a huge camp on a once-supposed evil site without the aid of military discipline, which have never been obtained in any military camp, and probably also in any camp of workmen abroad. Moreover, out of 350 visitors constantly in the health resort hotels last winter, on the remaining army sites of 1886, not a single case of typhoid occurred, and only one has originated in these hotels in the last five years. As regards, at least, Egypt, therefore, 'both these agencies (flies and dust) can only act where no proper precautions are taken.'

'This instance, sir, I think will help to minimise the difficulty (5) to which you have rightly drawn attention. I have shown how this Royal Water Corps, being responsible for the prevention of the diseases referred to, would take all steps to isolate these solitary cases at the earliest moment. I have ventured thus to trespass on your valuable space, feeling that as the plan I have suggested has been pronounced by a leading service paper as 'perfectly feasible,' we should at least do all in our power, in the medical profession, to assist the soldier of the future by obtaining for him pure or sterilised drinking water."

